# Long-term Effectiveness of a Smartphone App Combined With a Smart Band on Weight Loss, Physical Activity, and Caloric Intake in a Population With Overweight and Obesity (Evident 3 Study): Randomized Controlled Trial

**DOI:** 10.2196/30416

**Published:** 2022-02-01

**Authors:** Cristina Lugones-Sanchez, Jose I Recio-Rodriguez, Cristina Agudo-Conde, Irene Repiso-Gento, Esther G Adalia, José Ignacio Ramirez-Manent, Maria Antonia Sanchez-Calavera, Emiliano Rodriguez-Sanchez, Manuel A Gomez-Marcos, Luis Garcia-Ortiz, EVIDENT 3 Investigators

**Affiliations:** 1 Primary Care Research Unit of Salamanca (APISAL) Institute of Biomedical Research of Salamanca Health Service of Castilla y León Salamanca Spain; 2 Department of Nursing and Physiotherapy University of Salamanca Salamanca Spain; 3 Renedo de Esgueva Health Center Health Service of Castilla y León Valladolid Spain; 4 Health and Social Research Center University of Castilla-La Mancha Cuenca Spain; 5 Calvià Primary Care Center The Health Research Institute of the Balearic Islands Health Service of Balearic Islands Palma de Mallorca Spain; 6 Department of Medicine University of the Balearic Islands Palma de Mallorca Spain; 7 Las Fuentes Norte Health Center, Aragonese Group of Primary Care Research (GAIAP) Aragon Health Research Institute (IISA), Aragon Health Service Zaragoza Spain; 8 Department of Internal Medicine, Psychiatry and Dermatology University of Zaragoza Zaragoza Spain; 9 Department of Medicine, University of Salamanca Salamanca Spain; 10 Department of Biomedical and Diagnostic Sciences, University of Salamanca Salamanca Spain; 11 See Acknowledgements Barcelona Spain

**Keywords:** mobile app, telemedicine, eHealth, weight control, exercise, obesity, mobile phone

## Abstract

**Background:**

Multicomponent mobile health approaches can improve lifestyle intervention results, although little is known about their long-term effectiveness.

**Objective:**

This study aims to evaluate the long-term effectiveness (12 months) of a multicomponent mobile health intervention—combining a smartphone app, an activity tracker wristband, and brief counseling, compared with a brief counseling group only—on weight loss and improving body composition, physical activity, and caloric intake in Spanish sedentary adults with overweight or obesity.

**Methods:**

We conducted a randomized controlled, multicenter clinical trial (Evident 3). A total of 650 participants were recruited from 5 primary care centers, with 318 participants in the intervention group (IG) and 332 in the control group (CG). All participants were briefly counseled about a healthy diet and physical activity at the baseline visit. For the 3-month intervention period, the IG received training to use the app to promote healthy lifestyles and the smart band (Mi Band 2, Xiaomi). All measurements were performed at baseline and at 3 and 12 months. Physical activity was measured using the International Physical Activity Questionnaire–Short Form. Nutritional habits were assessed using the Food Frequency Questionnaire and Adherence to Mediterranean diet questionnaire.

**Results:**

Of the 650 participants included, 563 (86.6%) completed the 3-month visit and 443 (68.2%) completed the 12-month visit. After 12 months, the IG showed net differences in weight (−0.26, 95% CI −1.21 to 0.70 kg; *P*=.02), BMI (−0.06, 95% CI −0.41 to 0.28 points; *P*=.01), waist-height ratio (−0.25, 95% CI −0.94 to 0.44; *P*=.03), body adiposity index (−0.33, 95% CI −0.77 to 0.11; *P*=.03), waist circumference (−0.48, 95% CI −1.62 to 0.66 cm, *P*=.04) and hip circumference (−0.69, 95% CI –1.62 to 0.25 cm; *P*=.03). Both groups lowered daily caloric intake and increased adherence to the Mediterranean diet, with no differences between the groups. The IG increased light physical activity time (32.6, 95% CI −30.3 to 95.04 min/week; *P*=.02) compared with the CG. Analyses by subgroup showed changes in body composition variables in women, people aged >50 years, and married people.

**Conclusions:**

The low-intensity intervention of the Evident 3 study showed, in the IG, benefits in weight loss, some body composition variables, and time spent in light physical activity compared with the CG at 3 months, but once the devices were collected, the downward trend was not maintained at the 12-month follow-up. No differences in nutritional outcomes were observed between the groups.

**Trial Registration:**

ClinicalTrials.gov NCT03175614; https://clinicaltrials.gov/ct2/show/NCT03175614

**International Registered Report Identifier (IRRID):**

RR2-10.1097/MD.0000000000009633

## Introduction

### Background

The combination of excess body weight and physical inactivity contributes to global mortality [1,2] and to a shorter healthy life expectancy [3]. Moreover, both are associated with a major risk for serious chronic diseases, including type 2 diabetes and cardiovascular diseases, as well as increasing cardiovascular risk factors [4]. Such conditions may impact individual quality of life and well-being while increasing the burden on the health system [5]. Several strategies have been implemented to tackle obesity, mainly focusing on changing lifestyles. In general, weight loss interventions aim to increase physical activity, reduce daily energy intake, improve diet or nutritional habits, and achieve psychological changes [6]. Owing to the complex nature of obesity, multicomponent interventions, capable of addressing various aspects related to its causes, are shown to be more effective in reducing cardiovascular risk factors [7] and body weight [8]. However, finding accessible and cost-effective multicomponent strategies that promote healthy lifestyles over time is challenging.

### Mobile Health

Mobile health (mHealth) approaches, which are defined as the use of mobile wireless technologies for health [9], could optimize these efforts as portable and flexible tools, as well as improve the follow-up and feedback by registering health information [10] and provide efficient health management assistance for patients [11]. Specifically, some reviews suggested that mHealth could be more effective in losing weight than traditional approaches [12,13]. Among mHealth tools, smartphones are positioned as the most effective approach to achieve weight management [14], showing the most beneficial effects in the short term [15] (≤6 months). However, mobile phone intervention reports modest improvements in other lifestyles such as physical activity [16] or changes in biomarkers [13], highlighting the need to complement the intervention with other tools. Along these lines, wearable devices have garnered attention in improving physical activity and reducing sedentary lifestyle. Pedometer-based interventions have been widely explored [17], but the constant evolution of these tools requires the inclusion of emerging electronic devices [18]. Activity tracker wristbands, also called “smart bands,” have shown their validity and reliability in measuring physical activity outcomes [19] (eg, steps, kilometer walked, and intensity). Furthermore, a recent systematic review found that these device-based interventions are effective for increasing physical activity among chronic disease populations [20], making activity tracker wristbands a good option for inclusion in lifestyle interventions, as wearable activity trackers have the potential to increase physical activity participation [21]. However, wearable activity trackers alone may not be sufficient to achieve the expected lifestyle changes [22], so their inclusion in mHealth multicomponent obesity interventions, which appears to be more effective than app interventions alone [23], could be a beneficial strategy to obtain positive results in diet, physical activity, or other health variables [24,25]. In addition, self-monitoring in digital health interventions is associated with greater weight loss [26], so the use of both approaches could produce better weight outcomes.

Despite these promising results, the evidence for long-term efficacy is still limited, revealing that more evidence of effectiveness over long follow-up periods is required [27]. A systematic review showed that wearable devices might improve long-term physical activity and weight loss outcomes, although a comparison with traditional methods did not show clear benefits [28]. A similar situation exists with regard to multicomponent interventions with wearables, which may provide a strategy to improve long-term weight loss [29] despite the limited data on its effectiveness.

### Objectives

Previously, the Evident 2 study evaluated the effect of adding an app (Evident 2) to a standardized intervention designed to improve adherence to the Mediterranean diet and increase physical activity in the general population [30]. The Evident 3 study aimed to evaluate the long-term effectiveness (12 months) of a multicomponent mHealth intervention—combining a later version of the smartphone app (Evident 3) and a smart band, compared with a brief counseling–only group—on body weight loss, improving body composition, increasing time spent in physical activity, and decreasing caloric intake in Spanish sedentary adults with overweight and obesity. The short-term study results (3 months) related to body composition have already been published [31].

## Methods

### Design and Setting

A multicenter, randomized controlled clinical trial with 2 parallel groups was conducted in a primary care setting (Evident 3 study). The Primary Care Research Unit of Salamanca (APISAL) at the Biomedical Research Institute of Salamanca (IBSAL) coordinated the project in 5 health centers located in Spain from the Network for Preventive Activity and Health Promotion (REDIAPP; Salamanca, Valladolid, Cuenca, Palma de Mallorca, and Zaragoza). The main aim of the study was to evaluate the effect of the intervention on weight loss in participants with overweight and obesity [32] (ClinicalTrials.gov NCT03175614). Between June 2017 and June 2020, evaluations were made at baseline and after the completion of 3 and 12 months. The results presented in this paper correspond to the long-term effect (12 months) of the 3-month mHealth intervention on the primary outcome (weight loss) and secondary outcomes related to physical activity and diet.

### Study Population

The participants were selected by random sampling among patients who attended a consultation with their primary care provider in each participating center. The inclusion criteria were age between 20 and 65 years, a BMI between 27.5 kg/m^2^ and 40 kg/m^2^, classified as sedentary (20 minutes of vigorous-intensity activity ≤3 times per week; 30 minutes of moderate-intensity activity ≤5 times per week; or any combination of moderate and vigorous activity ≤5 times per week [33]), agreement to participate in the study, and signing the informed consent document. A detailed description of the exclusion criteria can be found elsewhere [32].

### Sample Size

The sample size calculation was performed for the primary study endpoint of weight loss at 12 months. Accepting an α risk of .05 and a β risk of .20, with an SD of 12 kg, estimated in participants from the Evident 2 study [30], 592 participants would be needed (296 per group) to detect a decrease in weight of ≥3 kg [34] in the intervention group (IG) versus the control group (CG), considering a 15% loss to follow-up. This effect size represents a 3% to 5% difference between groups, which should produce clinically relevant health benefits [35].

### Randomization

Participants were randomly assigned to either the IG or the CG after the baseline visit and provided informed consent. The allocation sequence was generated in a 1:1 ratio using the Epidat (version 4.2; Xunta de Galicia) software package [36] by an independent researcher and concealed until the group was assigned. Owing to the nature of the study, the intervention could not be blinded to the participants.

### Procedures

**Figure 1:**
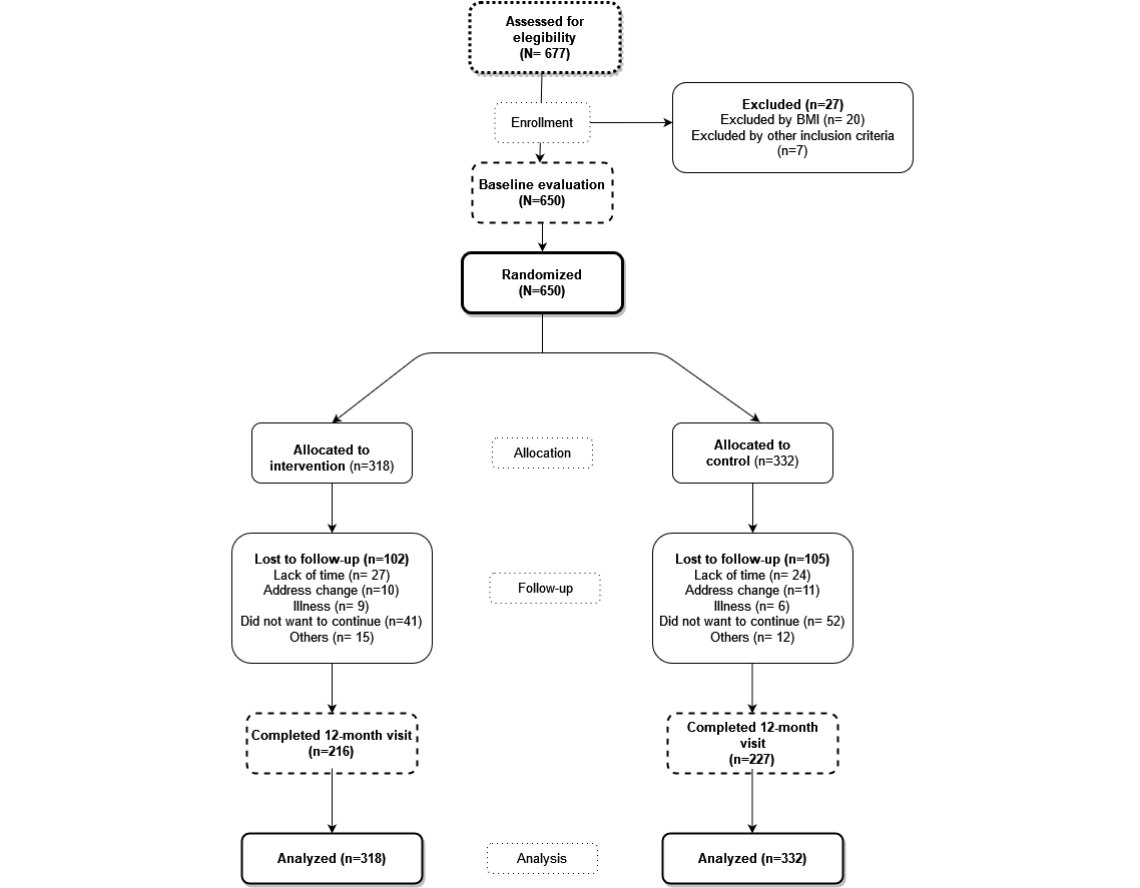
Flowchart of the Evident 3 study.

Each participant completed an initial visit and 2 follow-up visits at 3 and 12 months after randomization (Figure 1). They did not receive any compensation for the study or visit completion. Data from the visits were collected by a research nurse on a paper-based Case Report Form and recorded after the visit on the study website. The IG completed an additional appointment 7 days from the baseline visit to explain the use of the smartphone app and set it with the participant’s data. The researcher who carried out the additional appointment was different from the researcher who collected the data of the visits.

### Primary and Secondary Outcomes

#### Overview

The primary outcome was weight loss (kg). The secondary outcomes included changes in physical activity (min/week), caloric intake (kcal/day), adherence to the Mediterranean diet (points), and changes in body composition (anthropometric indexes). All outcomes were measured at baseline and at 3 and 12 months after randomization.

#### Weight Loss

Body weight was measured twice to the nearest 0.1 kg, with the participant barefoot and wearing light clothing, using a homologated electronic balance (Scale 7830; Soehnle Professional GmbH & Co). Height was measured with the participant barefoot in the standing position using a portable system (Seca 222; Medical scale and measurement system). BMI was calculated by dividing weight (kg) by height squared (m^2^). Waist circumference was measured twice on bare skin, using a flexible tape parallel to the floor, at the level of the upper border of the iliac crest, with the participant standing and after inspiration, following the recommendations of the Spanish Society for the Study of Obesity [37]. Hip circumference was similarly measured at the level of the trochanters over the underwear.

In addition, anthropometric indices were estimated to evaluate body composition changes:

Waist–height ratio =

WC / Height **(1)**

Body adiposity index =

[HC / (Height^1.5^)] − 18 **(2)**

Waist–hip ratio =

WC / HC **(3)**

Body shape index [38] =

WC / [(BMI^0.666^) × (Height ^0.5^)] **(4)**

Body roundness index [39] =

364.2 − 365.5 × √{1 − [WC / (2 × 3.1416)]^2^ / (0.5 × Height)^2^} **(5)**

#### Physical Activity

Physical activity was assessed using the short version of the International Physical Activity Questionnaire–Short Form (IPAQ-SF) [40]. The IPAQ-SF is a self-reported questionnaire that evaluates sitting and active time in the last 7 days through 7 questions. The categories consisted of walking, moderate-intensity activity, and vigorous-intensity activity, according to the energy expenditure estimated for each of them: 3.3, 4.0, and 8.0 metabolic equivalents (METs), respectively; 1 MET being the amount of oxygen consumed while sitting at rest [41]. Thus, the IPAQ-SF enables METs-minutes per week to be calculated, and participants were stratified into 3 activity levels (low, intermediate, and high).

#### Nutritional Habits

Caloric intake (kcal/day) and dietary habits of participants were evaluated using a semiquantitative Food Frequency Questionnaire previously validated in the Spanish population [42]. The frequency options are divided into 9 intake categories, ranging from never to >6 servings per day. Food Frequency Questionnaire data were used to estimate the daily intake of macro- and micronutrients and the mean kcal/day.

Adherence to the Mediterranean diet was assessed using the validated 14-point Mediterranean Diet Adherence Screener [43] developed by the prevention with Mediterranean diet study group, which comprises 12 questions on food consumption frequency and 2 questions on food intake habits. Each question was scored as 0 or 1, and the total score ranged from 0 to 14. A total score of 9 points or higher indicated adequate adherence.

#### Adherence to Self-monitoring on the Smartphone App

Adherence to self-monitoring on the smartphone app was assessed by the number of days that the participants logged into the app and recorded any dish or food. Records were classified into 4 categories: 0 days, 1 to 30 days, 31 to 60 days, and >60 days. Participants who used the app for >60 days during the 3 months that they had the app were classified as sufficiently adherent, whereas ≤60 days of use was classified as having low adherence.

#### Other Variables

##### Sociodemographic Variables

Data on age, sex, marital status, educational level, and occupation were collected at the time of inclusion in the study.

##### Peripheral Blood Pressure

Three measurements of systolic and diastolic blood pressure were performed using the average of the last 2 measurements with a validated Omron M10-IT model sphygmomanometer (Omron Healthcare). The measurements were made on both arms, with the participant seated, after at least 5 minutes of rest with an appropriately sized cuff, following the recommendations of the European Society of Hypertension [44].

##### Smoking Status

This was assessed through a questionnaire of 4 standard questions adapted from the World Health Organization monitoring of trends and determinants in cardiovascular disease study [45]. Study participants were classified as current smokers, former smokers (>1 year without smoking), or nonsmokers.

### Intervention

A detailed description of brief counseling and specific intervention has been published in the study protocol [32]. All the intervention materials were provided in Spanish.

#### Standard Counseling (CG and IG)

A trained nurse at each primary health center, who was not involved in other aspects of the study, gave 5 minutes of lifestyle counseling to both groups (CG and IG) before randomization, focusing on physical activity and diet in compliance with the international recommendations for the general population. The health benefits of physical activity were explained as well as the recommendation to complete at least 30 minutes of moderate activity 5 days a week, or 20 minutes of vigorous activity 3 days a week. Counseling on food was in compliance with the plate method [46], in which a plate is divided into 4 parts: half the plate for salad or vegetables, one-quarter for proteins (white meat preferred over red meat), and the final quarter for carbohydrates. In addition, a medium-sized piece of fruit and a skimmed dairy product should be consumed for dessert. This advice enhanced the intake of healthy food, according to the Mediterranean diet pattern, and daily caloric intake goals were not included. No reinforcement of counseling was offered at any other study visit or between the 3- and 12-month visits.

#### Specific Intervention (IG)

The IG received a low-intensity intervention consisting of a smartphone with the EVIDENT 3 app (Samsung Galaxy J3) and a smart band (Xiaomi Mi Band 2) for 3 months, corresponding to the length of the intervention, without any additional reinforcement or counseling by the investigators throughout the study. Participants were trained at another 15-minute visit scheduled 7 days after the baseline visit in the use of the app (EVIDENT 3 app [record entry no. 00/2017/2438]) specifically designed for the study by CGB Computer Company and APISAL, as well as the use of the smart band, instructing them to use both tools daily.

**Figure 2:**
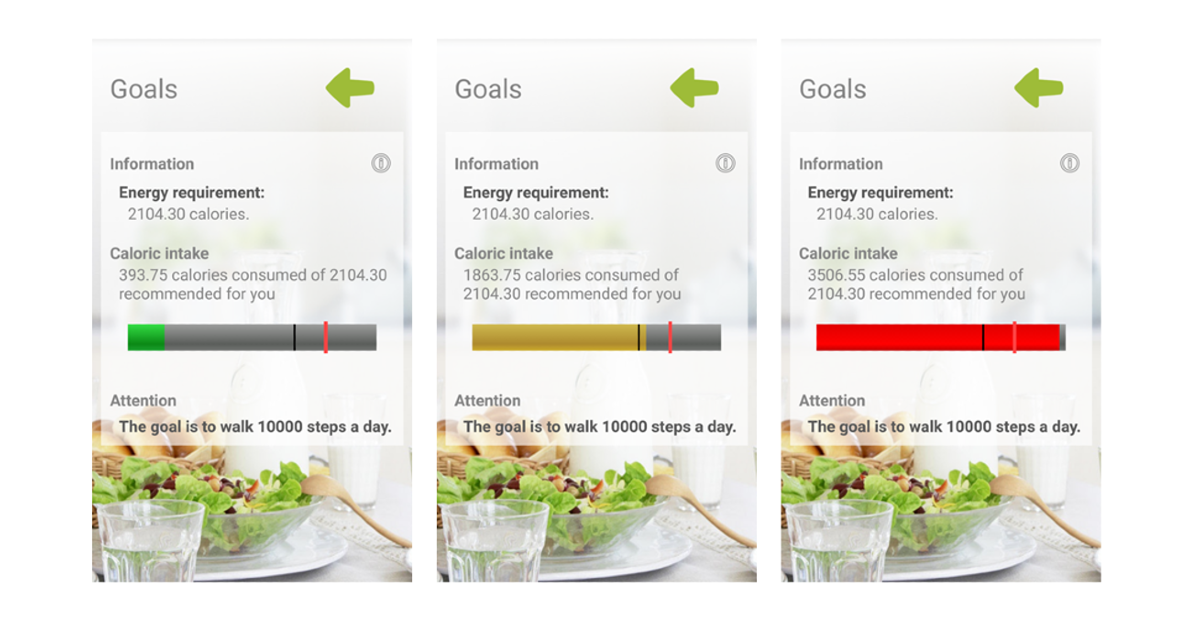
Evident 3 app screenshots.

During this visit, the app was configured with each participant’s data (sex, age, weight, and height). It was designed to allow full daily self-monitoring of food intake (Figure 2) and automatically record physical activity through the smart band, which was configured to synchronize with the app. Participants entered their food intake daily by selecting dishes and foods from the app menu and indicating the portion size. Food composition data were collected from the Spanish Food Composition Database [47], developed by Spanish Food Composition Database Network and Spanish Agency for Food Safety and Nutrition. Once all the daily information is collected, the app integrates the data to create personalized healthy food recommendations based on the Mediterranean diet pattern and specific targets for daily calorie intake that would lead to weight loss. The app displays the amount of calories recorded (Figure 2) and a bar that changes color (green, yellow, or red) according to the level set. It was configured to achieve a hypocaloric diet, calculating the upper limit (the red line) by adding, according to age and sex, the basal metabolic rate, diet-induced thermogenesis, and estimated energy expenditure for sedentary activities. The lower limit (the black line) was 85% of the calories calculated, and below this, the bar appeared in green; between the red and black lines, it appeared in yellow; and above the red line, it appeared in red. The participant was able to consult the app for these recommendations as well as information about caloric intake changes and macronutrient distribution (carbohydrates, proteins, and unsaturated and saturated fats). The smart band was set to congratulate the user when reaching 10,000 steps/day, and the app displayed this step recommendation in the goals section. Behavioral strategies were included in the mHealth intervention to enhance self-efficacy using self-monitoring, goal-setting, and positive reinforcement. At the 3-month visit, participants returned the intervention tools to the researchers. Thereafter, participants did not have access to the intervention devices and were advised not to use other digital tools for weight loss until the end of the study. All the information generated by the app was duly analyzed and entered into the database. In addition, once the tools were returned, monthly mean daily steps and activity minutes were collected for the last 2 of the 3 months of the intervention from the Mi Fit app (Xiaomi) to assess whether the smart band was worn.

### Blinding Strategy

The researcher who carried out the specific intervention was different from the person responsible for the assessment and the standard counseling; both were kept blinded throughout the study, as was the investigator who conducted the data analysis. Owing to the nature of the study, the participants could not be blinded. To prevent contamination between groups, in the follow-up visits (3 and 12 months), only the study variables were evaluated, but no advice or reinforcement was provided. In addition, the app was not available for download on the internet or anywhere until the end of the study, so the CG was not able to make use of it in any way. During the follow-up visits, participants were instructed not to use other digital health technologies.

### Statistical Analysis

Baseline characteristics of the study population were expressed as mean and SD for quantitative variables and as frequency distributions for categorical variables. Student *t* test (2-tailed), chi-square test, and Fisher exact test were used to determine differences in baseline characteristics between the IG and CG. Analyses of the results were performed on an intention-to-treat basis. Paired Student *t* test or McNemar test was applied to assess changes within the same group. To analyze the effect of the intervention, in the follow-up for primary and secondary endpoints, we performed several multivariate analyses of the variance of repeated measures using the general linear model, comparing the changes observed between the IG and CG in the analyzed variables, first unadjusted and then adjusted for age and sex.

We performed a subanalysis by multivariate analysis of the variance of repeated measures of the intervention effect in primary and secondary outcomes stratified by sex (men and women), age (<50 years and ≥50 years), and marital status (married, single, or others).

The contrast in the hypotheses established an α value of .05. The data were analyzed using SPSS Statistics software (version 26.0; IBM Corporation).

### Ethical Considerations

The study was approved by the clinical research ethics committee of the Health Area of Salamanca in April 2016. In addition, the study was approved by the ethics committees of the 4 collaborating centers: Aragón, Castilla-la Mancha, Baleares, and Valladolid Oeste. All procedures were performed in accordance with the ethical standards of the institutional research committee and the 2013 Declaration of Helsinki [48]. All participants signed written informed consent documents before participation in the study. The trial was registered at ClinicalTrials.gov with identifier NCT03175614 on May 31, 2017.

## Results

### Baseline Characteristics of the Participants and Follow-up

Of the 650 participants who completed the baseline visit, 563 (86.6%) completed the 3-month visit and 443 (68.2%) completed the 12-month visit. There were 207 (207/650, 31.8%) participants who dropped out of the study, 32.1% (102/318) in the IG and 31.6% (105/332) in the CG. Participants assigned to each group and the reasons for withdrawal from the trial may be consulted in Figure 1.

The clinical and sociodemographic baseline characteristics of the 650 participants are presented in Table 1. The mean age of the entire sample was 48.31 (SD 9.67) years, with a mean BMI of 33.0 (SD 3.48) kg/m^2^. In addition, 68.5% (445/650) of participants were women, 68.3% (444/650) were married, and 46.8% (304/650) were aged ≥50 years. No differences were observed between the study groups at baseline.

The comparison between the baseline characteristics of the 207 participants who dropped out and those who completed the study are shown in Multimedia Appendix 1. It should be noted that those who dropped out were younger (46.3 vs 49.2 years) and had a higher weight (93.3 vs 90.2 kg) and BMI (33.6 vs 32.7 kg/m^2^), with no difference in the rest of the variables analyzed.

**Table 1:** Baseline characteristics of study participants.

Characteristics		Intervention (n=318)	Control (n=332)
**Age (years)**			
	Value, mean (SD)	47.7 (10.1)	48.9 (9.2)
	<50, n (%)	178 (56)	168 (50.6)
	>50, n (%)	140 (44)	164 (49.4)
**Sex, n (%)**			
	Men	104 (32.7)	101 (30.4)
	Women	214 (67.3)	231 (69.6)
**Marital status, n (%)**			
	Single	60 (18.9)	74 (22.3)
	Married	222 (69.8)	222 (66.9)
	Separated	31 (9.7)	30 (9)
	Widower	5 (1.6)	6 (1.8)
**Employment status, n (%)**			
	Works outside of home	232 (72.9)	249 (75.1)
	Homemaker	22 (6.9)	21 (6.3)
	Retired	22 (6.9)	19 (5.7)
	Student	9 (2.8)	5 (1.5)
	Unemployed	33 (10.4)	38 (11.4)
**Educational level, n (%)**			
	University studies	122 (38.5)	134 (40.5)
	Middle or high school	158 (48.9)	152 (45.9)
	Elementary school	37 (11.7)	45 (13.6)
**Clinical variables, mean (SD)**			
	Weight (kg)	91.4 (14.8)	91.1 (14.8)
	BMI (kg/m^2^)	33.1 (3.4)	33.0 (3.6)
	Waist circumference (cm)	107.4 (12.9)	107.4 (10.7)
	Systolic blood pressure (mm Hg)	119 (15)	120 (16)
	Diastolic blood pressure (mm Hg)	79 (9)	81 (10)
	Heart ratio (bpm^a^)	72 (12)	74 (12)
	Total cholesterol (mg/dL)	198 (36)	202 (40)
	HDL^b^ cholesterol (mg/dL)	51 (13)	52 (12)
**BMI classification (kg/m^2^), n (%)**			
	27.5-29.9	75 (23.6)	82 (24.7)
	30-40	243 (76.4)	250 (75.3)
**Chronic diseases, n (%)**			
	Hypertension	88 (27.7)	116 (35.0)
	Dyslipidemia	73 (23.4)	87 (26.5)
	Diabetes mellitus	5 (1.7)	4 (1.3)
**Medication use, n (%)**			
	Antihypertensive drugs	50 (15.7)	69 (20.8)
	Lipid lowering drugs	50 (15.7)	56 (16.9)
	Hypothyroid drugs	31 (9.8)	37 (11.1)

^a^bpm: beats per minute.

^b^HDL: high-density lipoprotein.

### Adherence to Self-monitoring on the Smartphone App

Adherence to self-monitoring on the smartphone app was calculated from the app output data by an independent researcher. The median app use was 64.5 days out of the 90 days of the intervention (71.67%). Of the 318 participants assigned to the IG, 150 (47.2%) adhered sufficiently by recording data in the app between 61 and 90 days. In total, 3 participants did not register any food, and there were 36 data files from which no information was available for technical reasons (Multimedia Appendix 2). Multimedia Appendix 3 displays the median days of app use out of the 90 days in percentage to show adherence to self-monitoring on the app, grouped by sex, marital status, and age.

### Changes in Weight and Anthropometric Variables During the Study Period

Table 2 shows the decrease in body weight at 3 and 12 months in both groups. Comparing groups, the IG achieved a net weight loss difference of 0.76 (95% CI −1.33 to −0.19) kg at 3 months and 0.26 (95% CI −1.21 to 0.70; *P*=.02) kg at 12 months, more than the CG. The overall weight reduction of the IG was 2.05% at 3 months and 1.58% at 12 months, while the CG showed 1.1% and 1.26% reductions in body weight at 3 and 12 months, respectively. Only 18.2% (52/285) of the IG participants achieved a clinically significant weight loss of ≥5% at the 3 months visit and 19.4% (42/216) of them achieved that percentage at 12 months. In the CG, 12.9% (35/271) achieved a weight loss of 5% at 3 months and 18.5% (42/227) reached that loss at the 12-month visit. Regarding adherence to self-monitoring on the app, both in number of days used and median percentage of days, a positive correlation was found at 3 months with weight loss (*r*=0.239; *P*<.001) and BMI (*r*=0.203; *P*<.001) but not at 12 months or with weight (*r*=0.015; *P*=.83) and BMI (*r*=0.021; *P*=.77).

In addition, the IG showed changes in waist circumference (−0.76, 95% CI −1.47 to −0.05 cm) and hip circumference (−1.02, 95% CI −1.68 to −0.34 cm) compared with the CG at 3 months. Similar results were found at 12 months, with net decreases in waist circumference (−0.48, 95% CI −1.62 to 0.66 cm) and hip circumference (−0.69, 95% CI −1.62 to 0.25 cm; *P*=.04 and *P*=.03, respectively) between groups. Regarding body composition parameters, BMI decreased at 3 months (−0.30, 95% CI −0.52 to −0.09 points) and slightly changed at 12 months (−0.06, 95% CI −0.41 to 0.28 points; *P*=.01), comparing the study groups. Similar results were found for waist–height ratio at 3 months (−0.48, 95% CI −0.92 to −0.04) and 12 months (−0.25, 95% CI −0.94 to 0.44; *P*=.03) and body adiposity index at 3 months (−0.50, 95% CI −0.81 to −0.18) and 12-month follow-up (−0.33, 95% CI −0.77 to 0.11; *P*=.03) between groups. Although the IG tended to show decreases in the rest of the indexes analyzed (waist–hip ratio, body shape index, and body roundness index) at both follow-up visits, no significant differences were observed between groups.

**Figure 3:**
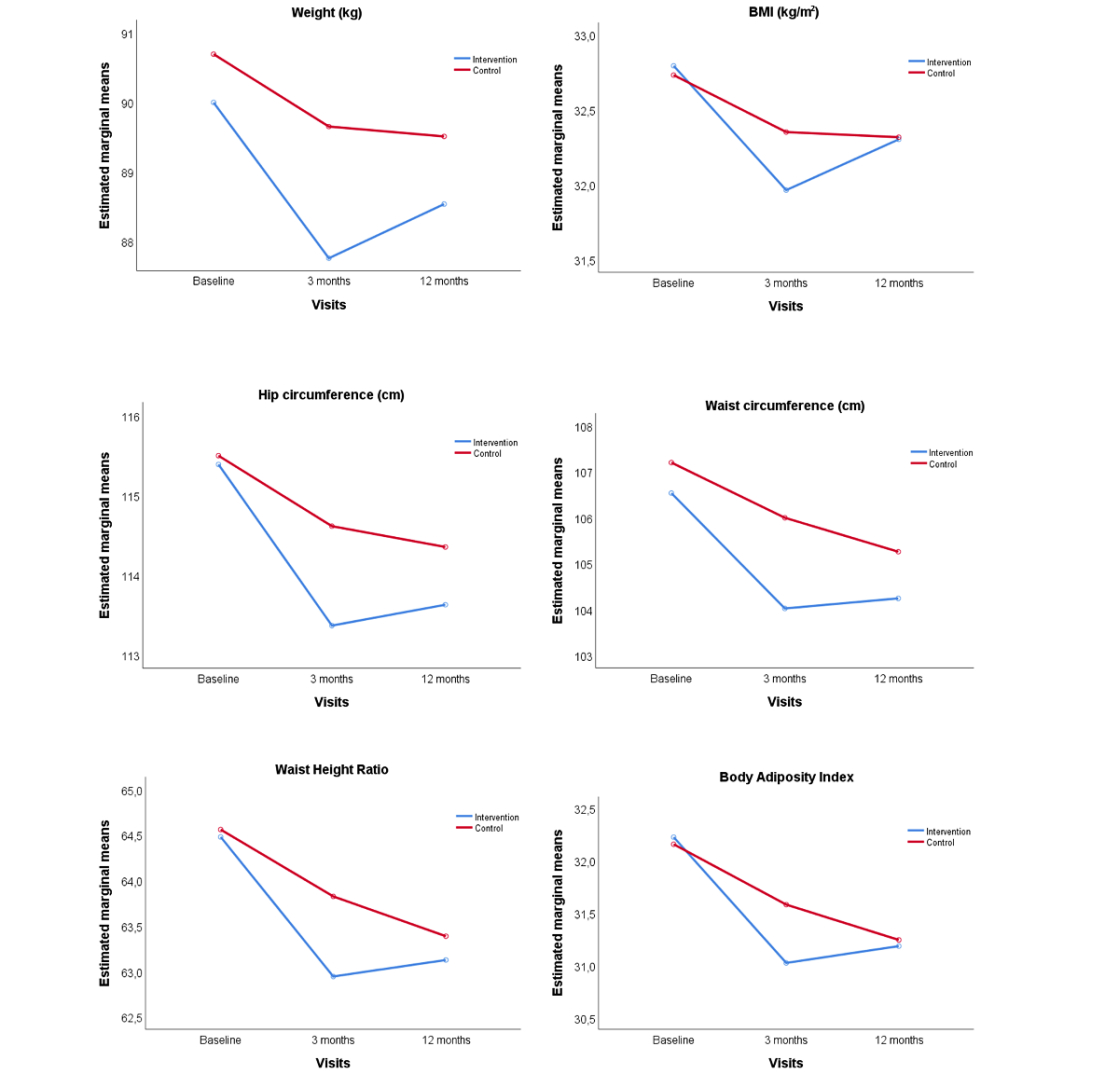
Evolution of weight, BMI, and other anthropometrics parameters from baseline to 3 and 12 months comparing the intervention and control group. *P* value between groups was adjusted by age and sex: weight, *P*=.02; waist circumference, *P*=.04; hip circumference, *P*=.03; BMI, *P*=.01; waist–height ratio, *P*=.03; and body adiposity index, *P*=.03.

Figure 3 shows the evolution of the main anthropometric parameters analyzed over time, with a decrease at 3 months in both groups, especially in the IG. However, the downward trend was not maintained in the IG at 12 months, while the CG continued to decrease.

**Table 2:** Effect of the mobile health intervention on body weight and other anthropometric parameters.

Parameters		Intervention group (n=318)			Control group (n=332)			Net difference			
		Values	*P* value	Values		*P* value	Values		*P* value^a^	*P* value^b^	
**Weight (kg)**											
	Baseline, mean (SD)	91.4 (14.8)	N/A^c^	91.1 (14.8)		N/A	N/A		N/A	N/A	
	3-month change, mean difference (95% CI)	−1.79 (−2.20 to −1.37)	<.001	−1.03 (−1.41 to −0.64)		<.001	−0.76 (−1.33 to −0.19)		N/A	N/A	
	12-month change, mean difference (95% CI)	−1.46 (−2.15 to −0.77)	<.001	−1.20 (−1.87 to −0.54)		<.001	−0.26 (−1.21 to 0.70)		.03	.02	
**Waist circumference (cm)**											
	Baseline, mean (SD)	107.4 (12.9)	N/A	107.4 (10.7)		N/A	N/A		N/A	N/A	
	3-month change, mean difference (95% CI)	−2.18 (−2.71 to −1.65)	<.001	−1.42 (−1.90 to −0.94)		<.001	−0.76 (−1.47 to −0.05)		N/A	N/A	
	12-month change, mean difference (95% CI)	−2.28 (−3.14 to −1.43)	<.001	−1.80 (−2.57 to −1.04)		<.001	−0.48 (−1.62 to 0.66)		.04	.04	
**Hip circumference (cm)**											
	Baseline, mean (SD)	116.4 (11.4)	N/A	115.5 (9.3)		N/A	N/A		N/A	N/A	
	3-month change, mean difference (95% CI)	−1.96 (−2.42 to −1.50)	<.001	−0.94 (−1.42 to −0.47)		<.001	−1.02 (−1.67 to −0.36)		N/A	N/A	
	12-month change, mean difference (95% CI)	−1.81 (−2.47 to −1.16)	<.001	−1.13 (−1.79 to −0.46)		.001	−0.69 (−1.62 to 0.25)		.03	.03	
**BMI (kg/m^2^)**											
	Baseline, mean (SD)	33.1 (3.4)	N/A	32.9 (3.6)		N/A	N/A		N/A	N/A	
	3-month change, mean difference (95% CI)	−0.69 (−0.85 to −0.53)	<.001	−0.38 (−0.52 to −0.24)		<.001	−0.30 (−0.52 to −0.09)		N/A	N/A	
	12-month change, mean difference (95% CI)	−0.49 (−0.74 to −0.24)	<.001	−0.43 (−0.66 to −0.19)		<.001	−0.06 (−0.41 to 0.28)		.02	.01	
**Waist–height ratio**											
	Baseline, mean (SD)	64.82 (6.9)	N/A	64.76 (5.7)		N/A	N/A		N/A	N/A	
	3-month change, mean difference (95% CI)	−1.35 (−1.68 to −1.02)	<.001	−0.87 (−1.16 to −0.58)		<.001	−0.48 (−0.92 to −0.04)		N/A	N/A	
	12-month change, mean difference (95% CI)	−1.34 (−1.86 to −0.83)	<.001	−1.09 (−1.56 to −0.63)		<.001	−0.25 (−0.94 to 0.44)		.04	.03	
**Body adiposity index**											
	Baseline, mean (SD)	36.76 (6.6)	N/A	36.31 (5.6)		N/A	N/A		N/A	N/A	
	3-month change, mean difference (95% CI)	−0.94 (−1.16 to −0.72)	<.001	−0.44 (−0.66 to −0.22)		<.001	−0.50 (−0.81 to −0.18)		N/A	N/A	
	12-month change, mean difference (95% CI)	−0.85 (−1.16 to −0.54)	<.001	−0.52 (−0.83 to −0.20)		.001	−0.33 (−0.77 to 0.11)		.03	.03	
**Waist–hip ratio**											
	Baseline, mean (SD)	0.9 (0.1)	N/A	0.9 (0.1)		N/A	N/A		N/A	N/A	
	3-month change, mean difference (95% CI)	0.00 (−0.01 to 0.00)	.07	0.00 (−0.01 to 0.00)		.02	0.00 (−0.01 to 0.01)		N/A	N/A	
	12-month change, mean difference (95% CI)	−0.01 (−0.01 to 0.00)	.13	−0.01 (−0.01 to 0.00)		.03	0.00 (−0.01 to 0.01)		.51	.46	
**Body shape index^d^**											
	Baseline, mean (SD)	8.1 (0.6)	N/A	8.1 (0.5)		N/A	N/A		N/A	N/A	
	3-month change, mean difference (95% CI)	−0.06 (−0.09 to −0.02)	.002	−0.04 (−0.08 to −0.01)		.01	−0.01 (−0.06 to 0.036)		N/A	N/A	
	12-month change, mean difference (95% CI)	−0.09 (−0.14 to −0.04)	<.001	−0.06 (−0.11 to −0.01)		.01	−0.03 (−0.09 to 0.043)		.52	.56	
**Body roundness index**											
	Baseline, mean (SD)	6.7 (1.9)	N/A	6.6 (1.4)		N/A	N/A		N/A	N/A	
	3-month change, mean difference (95% CI)	−0.32 (−0.40 to −0.24)	<.001	−0.21 (−0.28 to −0.14)		<.001	−0.11 (−0.21 to 0.00)		N/A	N/A	
	12-month change, mean difference (95% CI)	−0.31 (−0.43 to −0.18)	<.001	−0.26 (−0.37 to −0.14)		<.001	−0.05 (−0.22 to 0.12)		.07	.05	

^a^*P* value by analysis of variance.

^b^*P* value by analysis of variance adjusted by age and sex.

^c^N/A: not applicable.

^d^Body Round Shape results are displayed multiplied by 100 for easier reading.

### Changes in Diet and Physical Activity

In terms of diet, daily caloric intake was lower in both groups (IG: −295, 95% CI −391.63 to −198.93 kcal/day; CG: −222, 95% CI −310.62 to −135.14 kcal/day) at 12 months, with no significant differences between groups (Table 3). The Mediterranean diet adherence increased in both groups at 3 and 12 months, but the differences were not statistically significant. The IG experienced a trend toward increased adherence to the Mediterranean diet at 3 months, but decreased at 12 months, while adherence was maintained in the CG.

Physical activity time in all intensities (light, moderate, and vigorous) assessed by the IPAQ-SF increased in both groups at 3 and 12 months, whereas the time of sedentarism decreased. Although the IG tended to show greater increases in light activity, vigorous activity, and total activity time, only light physical activity (LPA) time showed a net increase of 81.2 (95% CI 31.93-130.58) minutes per week at 3 months and 32.6 (95% CI −30.31 to 95.04) minutes per week at 12 months (*P*=.02) compared with the CG. Regarding the smart band, Multimedia Appendix 4 shows the correlation between the IPAQ-SF data (METs per week and min/week for each activity level) at 3 months in the IG and the information collected from the smart band after 3 months of the intervention (daily average of steps and activity minutes), with a low correlation between variables, which was higher in women than in men.

**Table 3:** Effect of the mobile health intervention on diet and physical activity variables.

Variables		Intervention group (n=318)		Control group (n=332)		Net difference	
		Value	*P* value	Value	*P* value	Value	*P* value^a^
**Energy intake (kcal)**							
	Baseline, mean (SD)	2394.1 (676.4)	N/A^b^	2359.9 (681.1)	N/A	N/A	N/A
	3-month change, mean difference (95% CI)	−204.18 (−285.46 to −122.89)	<.001	−189.84 (−268.94 to −110.73)	<.001	−14.34 (−127.48 to 98.80)	N/A
	12-month change, mean difference (95% CI)	−295.28 (−391.63 to −198.93)	<.001	−222.88 (−310.62 to −135.14)	<.001	−72.40 (−202.13 to 57.33)	.59
**Score for adherence to MD^c^ (points)**							
	Baseline, mean (SD)	7.1 (1.9)	N/A	7.1 (1.9)	N/A	N/A	N/A
	3-month change, mean difference (95% CI)	0.56 (0.34 to 0.78)	<.001	0.41 (0.19 to 0.60)	<.001	0.15 (−0.15 to 0.45)	N/A
	12-month change, mean difference (95% CI)	0.37 (0.12 to 0.63)	.005	0.56 (0.29 to 0.82)	<.001	−0.19 (−0.56 to 0.17)	.06
**Score adherence to MD (≥9 points)**							
	Baseline, n (%)	81 (25.3)	N/A	84 (25.4)	N/A	N/A	N/A
	3-month change, percentage difference (95% CI)	8.04 (2.07 to 14.02)	.009	5.90 (0.22 to 11.59)	.04	2.14 (−6.11 to 10.39)	N/A
	12-month change, percentage difference (95% CI)	6.45 (−0.89 to 13.80)	.09	11.66 (4.74 to 18.58)	.001	−5.21 (−15.26 to 4.85)	.19
**Light activity (min/week)**							
	Baseline, mean (SD)	259.9 (283.4)	N/A	259.8 (287.9)	N/A	N/A	N/A
	3-month change, mean difference (95% CI)	102.85 (70.46 to 135.26)	<.001	21.60 (−15.96 to 59.16)	.26	81.26 (31.93 to 130.58)	N/A
	12-month change, mean difference (95% CI)	91.21 (58.76 to 125.65)	<.001	58.85 (6.24 to 111.46)	.03	32.36 (−30.31 to 95.04)	.02
**Moderate activity (min/week)**							
	Baseline, mean (SD)	53.0 (154.2)	N/A	41.3 (133.6)	N/A	N/A	N/A
	3-month change, mean difference (95% CI)	19.10 (−7.80 to 46.01)	.16	44.32 (22.06 to 66.57)	<.001	−25.21 (−60.27 to 9.85)	N/A
	12-month change, mean difference (95% CI)	2.04 (−28.58 to 32.65)	.90	38.90 (13.54 to 64.26)	.003	−36.86 (−76.39 to 2.66)	.06
**Vigorous activity (min/week)**							
	Baseline, mean (SD)	39.2 (228.0)	N/A	30.7 (143.2)	N/A	N/A	N/A
	3-month change, mean difference (95% CI)	20.80 (−1.26 to 42.85)	.06	13.73 (−15.81 to 42.26)	.36	7.07 (−29.44 to 43.58)	N/A
	12-month change, mean difference (95% CI)	25.29 (2.98 to 47.59)	.03	17.37 (−4.92 to 39.66)	.13	7.92 (−23.54 to 39.37)	.82
**Moderate to vigorous activity (min/week)**							
	Baseline, mean (SD)	92.2 (293.5)	N/A	71.9 (195.4)	N/A	N/A	N/A
	3-month change, mean difference (95% CI)	39.89 (5.07 to 74.73)	.03	58.04 (16.83 to 99.26)	.006	−18.15 (−71.77 to 35.48)	N/A
	12-month change, mean difference (95% CI)	27.33 (−15.38 to 70.03)	.21	56.27 (18.59 to 93.96)	.004	−28.95 (−85.63 to 27.73)	.33
**Total activity time (min/week)**							
	Baseline, mean (SD)	351.1 (415.8)	N/A	331.7 (345.2)	N/A	N/A	N/A
	3-month change, mean difference (95% CI)	142.75 (94.90 to 190.61)	<.001	79.65 (24.27 to 135.02)	.005	63.11 (−9.68 to 135.90)	N/A
	12-month change, mean difference (95% CI)	118.53 (60.59 to 176.48)	<.001	115.12 (45.01 to 185.23)	.001	3.41 (−87.63 to 94.46)	.57
**Total sitting time (min/week)**							
	Baseline, mean (SD)	2903.6 (1397.3)	N/A	2805.9 (1347.6)	N/A	N/A	N/A
	3-month change, mean difference (95% CI)	−174.98 (−294.58 to −55.40)	.004	−42.74 (−160.90 to 75.42)	.48	−132.24 (−300.17 to 35.70)	N/A
	12-month change, mean difference (95% CI)	−259.84 (−389.54 to −130.16)	<.001	−94.28 (−239.15 to 50.60)	.20	−165.57 (−359.74 to 28.60)	.09

^a^*P* value by analysis of variance.

^b^N/A: not applicable.

^c^MD: Mediterranean diet.

### Analyses of the Effect Stratified by Baseline Characteristics

Only women experienced decreases in weight, waist, and other anthropometric parameters analyzed, except for waist–hip ratio, body shape index, and body roundness index, with a net weight loss of 0.50 (95% CI −1.53 to 0.54; *P*=.002) kg at 12 months. With regard to age, those aged <50 years showed a decrease in hip circumference, while people aged >50 years showed a reduction in weight and BMI. Finally, analysis by marital status showed that only married people showed decreases in weight (−0.90, 95% CI −2.0 to 0.2 kg; *P*=.02), BMI (−0.3, 95% CI −0.7 to 0.1 points; *P*=.01), and other anthropometric parameters at 12 months. These analyses are presented in Multimedia Appendix 5.

## Discussion

### Principal Findings

#### Overview

The Evident 3 study evaluated the intervention effect and its maintenance over time at 3 and 12 months after the baseline visit. The main findings of the use of a smartphone app in combination with an activity tracker wristband for 3 months and brief counseling showed a greater weight loss compared with the CG, but once these devices were collected, the trend was not maintained at 12 months. Although both groups had reduced weight, BMI, and other anthropometric variables, the IG showed a greater trend to reduce weight, BMI, waist and hip circumference, waist–height ratio, and body adiposity index at the 3-month follow-up and did not maintain a downward trend at the 12-month visit. Regarding diet, both groups decreased their caloric intake (kcal) and improved their Mediterranean diet adherence, but no differences between groups were found. A similar result was observed in physical activity, where physical activity time increased in both groups, but the IG showed an increase in weekly LPA time at 12 months. Analyses stratified by baseline characteristics showed patterns toward greater changes in body composition variables in women, people aged >50 years, and married people.

#### Weight

Findings from meta-analyses have shown that mHealth weight loss interventions were effective in comparison with minimal intervention or control in the short term but with inconclusive long-term results [15,16]. The Evident 3 study provides insights into the long-term effects of weight loss, BMI, and other anthropometric variables at 3 months, but this trend was not maintained in the period when they no longer had access to the devices up to the 12-month visit. In addition, overall weight reduction in the IG was 2.05% at 3 months, so clinically relevant weight loss was not achieved (>5%).

Despite the heterogeneity found in these types of interventions, some systematic reviews [23,49] suggest that the combination of mHealth tools could be useful for changing lifestyles to healthier ones, but its effect on weight loss remains unclear. Along these lines, the Innovative Approaches to Diet, Exercise and Activity study, which provided a wearable technology combined with a website to the enhanced IG, found no significant difference in weight or physical activity between groups [29] across the 24-month intervention in young adults. Moreover, another study that evaluated the effect of a web-based weight loss program with and without an activity tracking device found that its addition to the intervention did not produce higher changes in weight loss at the 12-month follow-up [50] than those in the CG. In contrast, the Quant study, whose intervention included 3 feedback devices (Bioelectrical Impedance Analysis scale, blood pressure, and step counter) found a positive effect on fat loss at the 12-month follow-up [51]. These results are consistent with those found in our study, where there were differences between groups in weight, body composition variables, and physical activity but without reaching clinically relevant results.

Notwithstanding the effectiveness of self-monitoring behaviors in weight management [52], the challenge lies in finding a way to keep the users using the app, because the frequency of logging into the app is highly related to weight loss success in web-based interventions [53] and mHealth apps [52], as the user adopts new behaviors over time that is supported by the tools. In this regard, the rate of users who adhered sufficiently to the study app (150/318, 47.2%) may be insufficient to show better results in the main outcomes at 12 months. It should also be noted that between the period in which mHealth tools were collected at the 3-month follow-up and the 12-month visit, there was no reinforcement or any contact with the participants by the researchers. Therefore, despite not obtaining clinically relevant weight loss, the results suggest that the intervention should be modified (longer intervention period, improved adherence strategies, some professional support, etc), as the trend of weight reduction was not maintained over time when tools were removed. Moreover, the CG also reported weight loss but lower than that of the IG, which is commonly reported in weight loss interventions [25], especially if the CG received usual care [54].

#### Physical Activity and Diet

The study intervention, which included an activity tracker wristband, was shown to increase weekly LPA time measured by the IPAQ-SF. Increasing LPA may improve important health outcomes, such as markers of lipid and glucose metabolism and mortality in the general population [55]. Although the general physical activity recommendations are based on promoting moderate to vigorous physical activity, increasing LPA in the sedentary population may be a good starting point for decreasing inactivity in people with overweight and obesity [56]. Previous studies have reported beneficial changes in physical activity variables, observing a small increase in moderate to vigorous physical activity in women [57] and people with overweight and obesity [22] or in steps per day [20] in people with chronic diseases. Specifically, a recent study reported increased resistance training and reduced energy intake at 6 months using a multicomponent mHealth intervention [25], showing the potential of these tools in physical activity promotion by allowing a more tailored intervention and greater feedback.

Furthermore, energy intake was reduced, and adherence to the Mediterranean diet increased in both groups. However, the app intervention did not achieve better results than the CG. Potential explanations for this include that the brief diet counseling using the plate method was explained at the end of the baseline visit, which could lead to an improvement in the entire sample. Moreover, Solbrig et al [58] suggested that there is a mismatch between people’s need for help and what weight management apps provide, as people dislike counting calories, the basis of most self-management apps, and need more tailored support and motivational elements. Along these lines, a recent study found that a digital app that provides personalized nutritional recommendations appeared to be successful in reducing weight in users with obesity [59]. Future research will focus on the inclusion of new adaptive features in health apps to achieve greater results in health outcomes and higher rates of intervention adherence.

#### Analyses of the Effect Stratified by Baseline Characteristics

These analyses showed that the intervention was more effective in specific groups than in the general IG. Women showed greater weight loss, BMI changes, and other anthropometric variables, whereas men did not show differences. This could be explained by the higher rate of participation by women in this study (445/650, 68.5%), following the trend of weight management studies [60] and by the lower number of men included (n=205), which may have resulted in an underpowered analysis to find differences in the male group. In addition, women are more likely to participate in weight loss interventions [61] and use health apps [62], so the sex factor has to be considered. Regarding age, people aged >50 years obtained differences in more outcomes than younger people, and they were more adherent to the self-monitoring on the app. A systematic review found that middle-aged adults are more willing to adhere to such interventions with activity trackers [63] than younger people, so it is feasible that differences are found between age groups, as in the study results. In terms of marital status, married people seem to benefit more from the intervention. However, the unequal size of the groups could explain why single people, who obtained higher median adherence days than married people, did not show positive results. The influence of sociodemographic factors on the digital intervention effect, or adherence, has to be explored in-depth, but a study among users of eHealth approaches suggested that married people, among other characteristics, generally used more mHealth apps [64], which might lead to more positive results. Although more research is needed to determine which personal factors could influence mHealth effectiveness, these analyses highlight the need to develop more tailored interventions, adapting them according to certain characteristics of the user to enhance the effect of these digital approaches.

Finally, the average dropout rate was higher than expected (207/650, 31.8%) but balanced between study groups (IG: 102/318, 32.1% vs CG: 105/332, 31.6%). As a group, the participants who dropped out of the study were younger, with greater mean weight, BMI >30 kg/m^2^, and a higher proportion of smokers, in line with the dropout predictors found by a systematic review [65]. The participant attrition rate from digital interventions often exceeds 20% [66], and it is common to find large dropouts in weight management mobile phone apps [67] and multicomponent interventions [51]. Potential explanations could be participants’ higher weight loss expectations [65] and experiencing difficulty in maintaining self-monitoring.

### Strengths and Limitations

This study had several strengths. The study included a large sample with a multisite design with a wide range of ages and educational background, which offers robustness to the results obtained. Both the intervention and the statistical analysis were conducted by blinding researchers to the assignment groups. The 3- and 12-month visits allowed evaluation of the short-term and long-term effects of this type of technology on weight loss in the absence of additional face-to-face intervention. Moreover, the adherence rate to the self-monitoring diet on the app was acceptable (median percentage of days 71.7%).

In addition, some limitations of this study should be noted. Although participants were instructed not to use any other mHealth tool that could interfere with the study, there are no guarantees to ensure this occurred. The data collected from the smart band did not allow for the assessment of daily use and adherence to this device. In addition, the nature of the intervention precludes blinding of the participants, although recent findings suggest that blinding is less important than often believed [68]. The exposure time to the intervention (3 months) might not be sufficient to identify more positive results in changing lifestyles and weight loss. The number of participants in each baseline characteristics (sex, age, and marital status) group could be insufficient to show more relevant effects in the stratified analysis. Finally, the dropout rate of 31.8% (207/650) may have biased the final sample study composition and underpowered the study with regard to detecting a significant effect in the results between groups. However, random allocation and a balanced dropout between arms show that the group characteristics differ little from the initial sample, making the comparison between groups possible [69].

### Conclusions

The low-intensity intervention of the Evident 3 study showed in the IG, benefits on weight loss, some body composition variables, and time spent in LPA compared with the CG at 3 months, but once the devices were collected, the downward trend was not maintained at the 12-month follow-up. No differences in nutritional outcomes were observed between the groups. Analyses stratified by baseline characteristics revealed that the intervention was more effective in women, people aged ≥50 years, and married participants. Further research is needed to determine the optimum intervention period to achieve greater results, as well as the inclusion of more tailored strategies in health apps and weight management interventions that improve intervention adherence and retention rates.

## Appendix

Multimedia Appendix 1 Baseline characteristics comparison between participants who completed the study and those who dropped out.

Multimedia Appendix 2 Adherence to the smartphone app (number of days with a record in the app).

Multimedia Appendix 3 Adherence to self-monitoring on the app analyzed by the median percentage out of the 90 days of the intervention grouped by sex, marital status and age.

Multimedia Appendix 4 Correlations between International Physical Activity Questionnaire-Short Form and the Smart band at the 3-month visit in the intervention group.

Multimedia Appendix 5 Analysis of the mobile health intervention effect on weight and body composition variables grouped by baseline characteristics.

Multimedia Appendix 6 CONSORT eHEALTH Checklist (V 1.6.1).

## Abbreviations

APISAL: Primary Care Research Unit of Salamanca
CG: control group
IBSAL: Biomedical Research Institute of Salamanca
IG: intervention group
IPAQ-SF: International Physical Activity Questionnaire–Short Form
LPA: light physical activity
MET: metabolic equivalent
mHealth: mobile health
REDIAPP: Network for Preventive Activity and Health Promotion
