# Supplementary material for: Long-term Effectiveness of a Smartphone App Combined With a Smart Band on Weight Loss, Physical Activity, and Caloric Intake in a Population With Overweight and Obesity (Evident 3 Study): Randomized Controlled Trial
Source: J Med Internet Res. 2022 Feb 1;24(2):e30416. doi: 10.2196/30416 (PMC8848250; doi:10.2196/30416)
Supplement: Multimedia Appendix 1 [file jmir_v24i2e30416_app1.pdf]

Table S1. Baseline characteristics comparison between participants who completed the study and those who dropped out

	**Complete**<br>n = 443<br>Mean	**Loss of follow-up**<br>n = 207<br>Mean	*P* value
Age in years, mean (SD)	49.2 (9.4)	46.3 (9.9)	<.001
**Sex , n (%)**			
Men	140 (31.6)	65 (31.4)	.96
Women	303 (68.4)	142 (68.6)	
**Smoker status, n (%)**			
Non-smoker	181 (40.9)	82 (39.6)	.40
Current smoker	91 (20.5)	52 (25.1)	
Former smoker	171 (38.6)	73 (35.3)	
**Clinical variables, mean (SD)**			
Weight, kg	90.2 (14.0)	93.3 (16.2)	.02
BMI, kg/m^2	32.7 (3.4)	33.6 (3.6)	.003
Waist circumference, cm	106.7 (10.5)	108.8 (14.3)	.06
Systolic blood pressure, mmHg	119.6 (15.9)	119.9 (14.7)	.85
Diastolic blood pressure, mmHg	80.4 (9.8)	79.5 (9.7)	.27
Total Cholesterol, mg/dl	200.0 (39.1)	199.4 (34.7)	.86
HDL Cholesterol, mg/dl	51.6 (12.6)	51.3 (12.0)	.73
**BMI classification, n (%)**			
27.5-29.9	117 (26.4)	40 (19.3)	.05
30-40	326 (73.6)	167 (80.7)	
**Chronic diseases, n (%)**			
Hypertension	144 (32.5)	60 (29.1)	.41
Dyslipidaemia	122 (27.5)	38 (19.3)	.03
Diabetes Mellitus	5 (1.2)	4 (2.2)	.47
**Marital status, n (%)**			
Single	94 (21.2%)	40 (19.3)	.11
Married	299 (67.5%)	145 (70.0)	
Separated	39 (8.8%)	22 (10.6)	
Widower	11 (2.5%)	0 (0.0)	
**Employment status, n (%)**			
Works outside of home	322 (72.7)	159 (76.8)	.68
Homemaker	32 (7.2)	11 (5.3)	
Retired	30 (6.8)	11 (5.3)	
Student	11 (2.5)	3 (1.4)	
Unemployed	48 (10.8)	23 (11.1)	
**Educational level, n (%)**			
University studies	178 (40.4)	78 (37.7)	.70
Middle or High school	210 (47.6)	100 (48.3)	
Elementary school	49.2 (9.4)	46.3 (9.9)	
