# Supplementary figures and images for: Long-term Effectiveness of a Smartphone App Combined With a Smart Band on Weight Loss, Physical Activity, and Caloric Intake in a Population With Overweight and Obesity (Evident 3 Study): Randomized Controlled Trial
Source: J Med Internet Res. 2022 Feb 1;24(2):e30416. doi: 10.2196/30416 (PMC8848250; doi:10.2196/30416)

**Figure S1.** Adherence to the smartphone app (number of days with a record in the app).

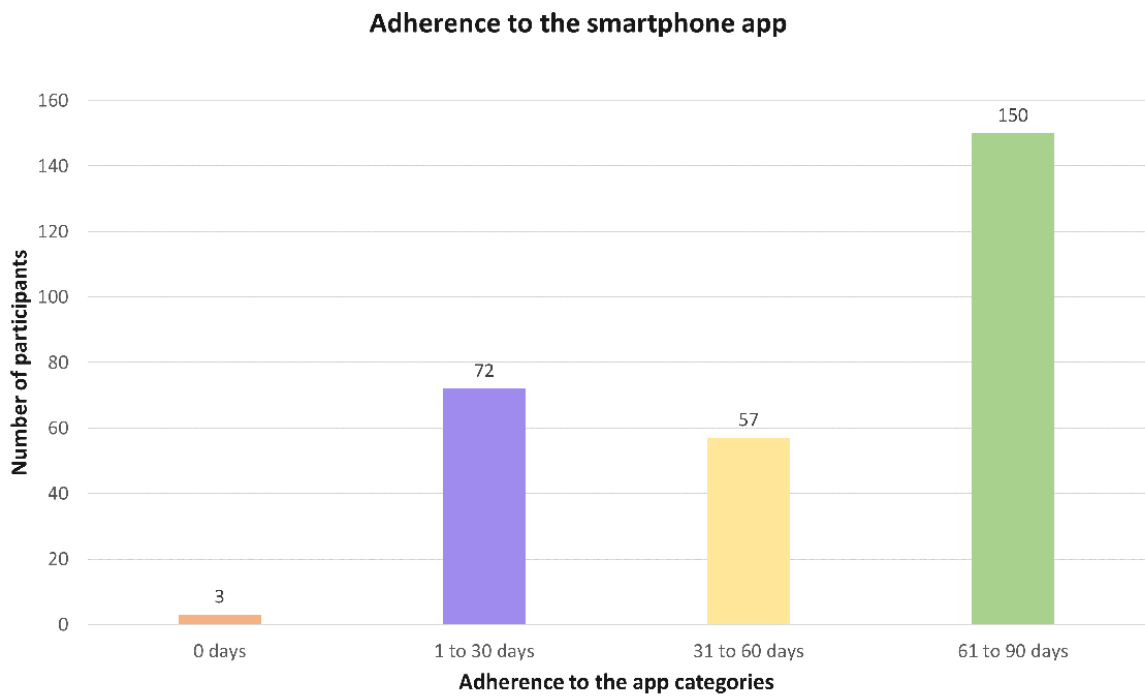

Supplement: Multimedia Appendix 2 [file jmir_v24i2e30416_app2.pdf]
