# Supplementary material for: Long-term Effectiveness of a Smartphone App Combined With a Smart Band on Weight Loss, Physical Activity, and Caloric Intake in a Population With Overweight and Obesity (Evident 3 Study): Randomized Controlled Trial
Source: J Med Internet Res. 2022 Feb 1;24(2):e30416. doi: 10.2196/30416 (PMC8848250; doi:10.2196/30416)
Supplement: Multimedia Appendix 3 [file jmir_v24i2e30416_app3.pdf]

Table S2. Adherence to self-monitoring on the app analyzed by the median percentage out of the 90 days of the intervention grouped by sex, marital status and age

Intervention group		Percentage of days of app use (Median)	*P*-value
**Sex**			
	Women	70.00	.38
	Men	75.56	
**Age**			
	≤ 50 years	57.51	.001
	> 50 years	84.44	
**Marital status**			
	Single	82.22	.10
	Married	73.89	
	Divorced	43.33	
	Widower	42.22	
