# Supplementary material for: Long-term Effectiveness of a Smartphone App Combined With a Smart Band on Weight Loss, Physical Activity, and Caloric Intake in a Population With Overweight and Obesity (Evident 3 Study): Randomized Controlled Trial
Source: J Med Internet Res. 2022 Feb 1;24(2):e30416. doi: 10.2196/30416 (PMC8848250; doi:10.2196/30416)
Supplement: Multimedia Appendix 4 [file jmir_v24i2e30416_app4.pdf]

Table S3. Correlations between IPAQ-SF and the Smart band at the 3-month visit in the IG

	Smart band					
	**Total**		**Men**		**Women**	
IPAQ-SF	**Steps/day**	**Min/day**	**Steps/day**	**Min/day**	**Steps/day**	**Min/day**
**METS/min/week**	0.372[b]	0.322[b]	0.337[b]	0.302[b]	0.399[b]	0.337[b]
**Min/week/sedentary**	-0.266[b]	-0.299[b]	-0.255[a]	-0.278[a]	-0.359[b]	-0.407[b]
**Min/week/MVPA**	0.154[a]	0.145[a]	0.169	0.181	0.115	0.086
**Min/week/Total activity**	0.390[b]	0.337[b]	0.375[b]	0.331[b]	0.413[b]	0.355[b]

Abbreviations: Min: Minutes; MVPA: Moderate-vigorous physical activity

[a]Correlation is significant at the P = .01 level (2-tailed).

[b]Correlation is significant at the P = .05 level (2-tailed).
