# Supplementary material for: Long-term Effectiveness of a Smartphone App Combined With a Smart Band on Weight Loss, Physical Activity, and Caloric Intake in a Population With Overweight and Obesity (Evident 3 Study): Randomized Controlled Trial
Source: J Med Internet Res. 2022 Feb 1;24(2):e30416. doi: 10.2196/30416 (PMC8848250; doi:10.2196/30416)
Supplement: Multimedia Appendix 5 [file jmir_v24i2e30416_app5.pdf]

Table S4. Analysis of the mHealth intervention effect on weight and body composition variables grouped by baseline characteristics

	Baseline visit (IG)	Baseline visit (CG)	3-month Net difference	12-month Net difference	
Sex	Mean (SD)	Mean (SD)	Mean (CI 95%)	Mean (CI 95%)	*P* value
**Men**					
Weight, kg	101.5 (15.0)	102.0 (12.7)	0.25 (-0.88 to 1.39)	0.25 (-1.78 to 2.29)	.86
Waist circumference, cm	112.9 (15.5)	112.5 (8.7)	-0.74 (-1.88 to 0.41)	-0.50 (-2.85 to 1.85)	.57
Hip circumference, cm	113.9 (14.0)	112.8 (7.6)	-0.45 (-1.55 to 0.65)	0.21 (-1.45 to 1.86)	.51
BMI, kg/m^2	33.1 (3.7)	33.4 (3.3)	0.06 (-0.32 to 0.44)	0.05 (-0.62 to 0.72)	.90
Waist height Ratio	64.5 (8.1)	64.5 (5.0)	-0.45 (-1.11 to 0.22)	-0.33 (-1.67 to 1.01)	.54
Body adiposity index	31.2 (5.5)	30.9 (3.1)	-0.22 (-0.70 to 0.27)	0.06 (-0.64 to 0.67)	.52
Waist hip ratio	1.0 (0.1)	1.0 (0.1)	0.00 (-0.01 to 0.01)	-0.01 (-0.02 to 0.01)	.78
Body shape index	0.1 (0.0)	0.1 (0.0)	0.00 (0.00 to 0.00)	0.00 (0.00 to 0.00)	.33
Body roundness index	6.6 (2.4)	6.5 (1.2)	-0.09 (-0.25 to 0.08)	-0.06 (-0.40 to 0.27)	.64
**Women**					
Weight, kg	86.5 (12.0)	86.3 (13.1)	-1.23 (-1.87 to -0.60)	-0.50 (-1.53 to 0.54)	.002
Waist circumference, cm	104.7 (10.5)	105.1 (10.8)	-0.78 (-1.68 to 0.12)	-0.47 (-1.75 to 0.81)	.048
Hip circumference, cm	117.6 (9.7)	116.7 (9.8)	-1.29 (-2.11 to -0.47)	-1.10 (-2.24 to 0.04)	.02
BMI, kg/m^2	33.1 (3.3)	32.8 (3.7)	-0.48 (-0.73 to -0.22)	-0.12 (-0.52 to 0.28)	.003
Waist height Ratio	65.0 (6.3)	64.9 (5.9)	-0.51 (-1.07 to 0.06)	-0.22 (-1.02 to 0.58)	.05
Body adiposity index	39.5 (4.6)	38.6 (4.7)	-0.63 (-1.04 to -0.23)	-0.52 (-1.08 to 0.04)	.02
Waist hip ratio	0.9 (0.1)	0.9 (0.1)	0.00 (0.00 to 0.01)	0.00 (-0.01 to 0.01)	.31
Body shape index	0.1 (0.0)	0.1 (0.0)	0.00 (0.00 to 0.00)	0.00 (0.00 to 0.00)	.91
Body roundness index	6.7 (1.6)	6.6 (1.5)	-0.12 (-0.25 to 0.02)	-0.04 (-0.24 to 0.15)	.07
Age					
**< 50 age**					
Weight, kg	93.0 (16.0)	94.3 (15.4)	-0.33 (-1.13 to 0.47)	-0.34 (-1.75 to 1.07)	.51
Waist circumference, cm	107.3 (14.8)	108.0 (11.7)	-0.90 (-1.91 to 0.11)	-1.04 (-2.72 to 0.65)	.10
Hip circumference, cm	117.1 (12.3)	117.6 (9.7)	-1.23 (-2.19 to -0.26)	-0.99 (-2.32 to 0.35)	.03
BMI, kg/m^2	33.2 (3.5)	33.6 (3.7)	-0.17 (-0.47 to 0.14)	-0.11 (-0.61 to 0.39)	.43
Waist height Ratio	64.3 (7.5)	64.6 (6.2)	-0.59 (-1.21 to 0.03)	-0.62 (-1.64 to 0.39)	.09
Body adiposity index	36.6 (6.1)	36.7 (5.9)	-0.60 (-1.67 to -0.14)	-0.48 (-1.10 to 0.14)	.03
Waist hip ratio	0.9 (0.1)	0.9 (0.1)	0.00 (-0.01 to 0.01)	0.00 (-0.01 to 0.01)	.75
Body shape index	0.1 (0.0)	0.1 (0.0)	0.00 (0.00 to 0.00)	0.00 (0.00 to 0.00)	.14
Body roundness index	6.5 (2.1)	6.6 (1.5)	-0.12 (-0.27 to 0.03)	-0.13 (-0.39 to 0.12)	.15
**> 50 age**					
Weight, kg	89.4 (12.9)	87.7 (13.4)	-1.26 (-2.07 to -0.46)	-0.13 (-1.45 to 1.18)	.01
Waist circumference, cm	107.6 (10.1)	106.8 (9.6)	-0.60 (-1.62 to 0.42)	0.20 (-1.35 to 1.74)	.22
Hip circumference, cm	115.4 (10.1)	113.4 (8.5)	-0.77 (-1.68 to 0.14)	-0.30 (-1.61 to 1.01)	.49
BMI, kg/m^2	33.0 (3.4)	32.3 (3.3)	-0.46 (-0.77 to -0.16)	-0.01 (-0.48 to 0.47)	.008

Waist height Ratio	65.5 (6.1)	64.9 (5.1)	-0.36 (-0.99 to 0.26)	0.19 (-0.75 to 1.12)	.19
Body adiposity index	36.9 (6.3)	39.9 (5.2)	-0.37 (-0.80 to 0.06)	-0.15 (-0.77 to 0.48)	.49
Waist hip ratio	0.9 (0.1)	0.9 (0.1)	0.00 (-0.01 to 0.01)	0.00 (-0.01 to 0.02)	.57
Body shape index	0.1 (0.0)	0.1 (0.0)	0.00 (0.00 to 0.00)	0.00 (0.00 to 0.00)	.88
Body roundness index	6.8 (1.5)	6.6 (1.3)	-0.09 (-0.24 to 0.07)	0.05 (-0.17 to 0.28)	.20
Marital status					
**Single**					
Weight, kg	90.1 (14.2)	93.0 (18.1)	0.04 (-1.25 to 1.33)	0.75 (-1.64 to 3.14)	.32
Waist circumference, cm	104.9 (11.0)	109.5 (13.4)	0.94 (-0.95 to 2.83)	0.78 (-2.26 to 3.82)	.58
Hip circumference, cm	116.0 (8.9)	117.7 (10.4)	-0.57 (-2.10 to 0.96)	-0.51 (-2.80 to 1.77)	.74
BMI, kg/m^2	32.7 (3.5)	33.3 (3.9)	-0.02 (-0.58 to 0.55)	0.40 (-0.48 to 1.28)	.27
Waist height Ratio	63.4 (6.4)	65.8 (6.4)	0.51 (-0.68 to 1.71)	0.55 (-1.30 to 2.40)	.54
Body adiposity index	36.7 (5.5)	37.0 (5.2)	-0.31 (-1.05 to 0.42)	-0.26 (-1.33 to 0.81)	.72
Waist hip ratio	0.9 (0.1)	0.9 (0.1)	0.01 (0.00 to 0.03)	0.01 (-0.02 to 0.04)	.42
Body shape index	0.1 (0.0)	0.1 (0.0)	0.00 (0.00 to 0.00)	0.00 (0.00 to 0.00)	.92
Body roundness index	6.3 (1.6)	6.9 (1.6)	0.14 (-0.14 to 0.43)	0.16 (-0.30 to 0.62)	.497
**Married**					
Weight, kg	92.2 (15.4)	90.4 (13.8)	-1.09 (-1.75 to -0.42)	-0.90 (-2.00 to 0.20)	.02
Waist circumference, cm	108.1 (13.7)	106.8 (9.8)	-1.29 (-2.08 to -0.50)	-1.21 (-2.51 to 0.08)	.01
Hip circumference, cm	116.4 (12.4)	114.9 (9.1)	-1.27 (-2.06 to -0.48)	-1.16 (-2.24 to -0.07)	.009
BMI, kg/m^2	33.1 (3.4)	32.8 (3.5)	-0.43 (-0.67 to -0.18)	-0.33 (-0.72 to 0.06)	.01
Waist height Ratio	65.0 (7.1)	64.5 (5.4)	-0.81 (-1.29 to -0.33)	-0.72 (-1.50 to 0.06)	.01
Body adiposity index	36.5 (6.4)	36.1 (5.6)	-0.61 (-0.99 to -0.23)	-0.56 (-1.08 to- 0.05)	.007
Waist hip ratio	0.9 (0.1)	0.9 (0.1)	0.00 (-0.01 to 0.00)	0.00 (-0.01 to 0.01)	.64
Body shape index	0.1 (0.0)	0.1 (0.0)	0.00 (0.00 to 0.00)	0.00 (0.00 to 0.00)	.34
Body roundness index	6.7 (2.0)	6.6 (1.3)	-0.18 (-0.30 to -0.07)	-0.17 (-0.36 to 0.03)	.02
**Others**					
Weight, kg	89.1 (12.1)	90.9 (13.1)	-0.20 (-2.16 to 1.75)	1.96 (-1.07 to 5.00)	.28
Waist circumference, cm	107.2 (10.5)	106.2 (9.2)	-0.76 (-3.14 to 1.61)	1.57 (-1.81 to 4.95)	.28
Hip circumference, cm	116.6 (9.0)	115.2 (7.3)	-0.41 (-2.43 to 1.62)	1.80 (-1.15 to 4.75)	.29
BMI, kg/m^2	33.6 (3.4)	32.9 (3.4)	-0.12 (-0.78 to 0.54)	0.76 (-0.32 to 1.84)	.17
Waist height Ratio	66.0 (6.7)	64.0 (5.4)	-0.46 (-1.89 to 0.97)	1.02 (-0.99 to 3.04)	.21
Body adiposity index	38.4 (6.4)	36.1 (6.3)	-0.18 (-1.14 to 0.78)	0.92 (-0.46 to 2.31)	.22
Waist hip ratio	0.9 (0.1)	0.9 (0.1)	0.00 (-0.03 to 0.02)	0.00 (-0.02 to 0.02)	.44
Body shape index	0.1 (0.0)	0.1 (0.0)	0.00 (0.00 to 0.00)	0.00 (0.00 to 0.00)	.75
Body roundness index	6.9 (1.7)	6.4 (1.3)	-0.11 (-0.46 to 0.23)	0.26 (-0.23 to 0.74)	.18

Abbreviations: IG: Intervention group; CG: Control group;
